# Evaluation of Psychometric Proprieties of Mann Assessment of Swallowing Ability (MASA) in Italian Language: A Cross-Sectional Study

**DOI:** 10.3390/brainsci14090900

**Published:** 2024-09-05

**Authors:** Arianna Sterbini, Patrizia Marroni, Annamaria Servadio, Giulia Rossi, Anna Berardi, Rachele Simeon, Giovanni Galeoto

**Affiliations:** 1 School of Speech and Language Therapy, University of Rome Tor Vergata, 00133 Rome, Italy; arianna.sterbini@alumni.uniroma2.eu (A.S.); patrizia.marroni@ptvonline.it (P.M.); 2 Department of Health Professions, University of Rome Tor Vergata, 00133 Rome, Italy; servadio@med.uniroma2.it; 3 Department of Human Neurosciences, Sapienza University of Rome, 00185 Rome, Italy; giu.rossi@uniroma1.it (G.R.); rachele.simeon@uniroma1.it (R.S.); giovanni.galeoto@uniroma1.it (G.G.); 4 IRCCS Neuromed, Pozzilli, 86077 Isernia, Italy

**Keywords:** assessment, dysphagia, psychometric properties, MASA

## Abstract

The aim of this cross-sectional study was to translate, culturally adapt, and evaluate the psychometric properties of the Italian version of the Mann Assessment of Swallowing Ability (MASA) in post-stroke individuals. The original MASA scale was translated and culturally adapted from English to Italian following the international guidelines. The internal consistency and test–retest reliability of the MASA-IT were assessed, and its concurrent validity was examined through Pearson correlation coefficients with the Italian versions of two established gold standard scales for dysphagia assessment: the Dysphagia Outcome Severity Scale (DOSS) and the De Pippo Test—Three Oz Water Swallow. The MASA-IT was administered to 78 participants. The items demonstrated excellent internal consistency, with Cronbach’s alpha ranging between 0.86 and 0.89. The interclass correlation coefficient was 0.98 for inter-rater reliability and 0.99 for intra-rater reliability, indicating high reproducibility. Regarding concurrent validity, the MASA-IT showed a strong direct correlation with the DOSS scale (r = 0.949, *p* = 0.01) and an inverse correlation with the De Pippo Test (r = −0.783, *p* = 0.01), confirming its good concurrent validity. The Italian version of the Mann Assessment of Swallowing Ability (MASA-IT) is a reliable and valid tool for assessing swallowing ability in post-stroke patients. Its strong psychometric properties make it well suited for clinical use in Italy.

## 1. Introduction

Stroke remains a significant public health issue in Italy, with approximately 200,000 new cases occurring annually. Among these, 80% are new incidents and 20% are relapses, highlighting the chronic nature of the condition. Stroke is a leading cause of disability, particularly affecting individuals over the age of 65 [1]. One of the prevalent complications associated with acute stroke is dysphagia, affecting about 50% of stroke patients [2]. This symptom is not only common but also critical, as it can lead to severe complications, including malnutrition, aspiration pneumonia, and increased mortality rates [3].

Dysphagia, or difficulty swallowing, presents a major challenge in stroke management due to its potential to cause life-threatening conditions and diminish patients’ quality of life. According to the “Linee Guida Sulla Gestione Del Paziente Disfagico Adulto in Foniatria E Logopedia”, dysphagia can severely impact self-esteem, safety, and work capacity, leading to significant declines in overall well-being [2]. The presence of dysphagia poses a considerable public health challenge, affecting both individual health outcomes and healthcare costs.

Early and effective screening for dysphagia is crucial for managing the condition, as it facilitates the identification of at-risk patients and prompts timely professional assessments. Although screening tools do not provide definitive diagnoses, they are essential for early intervention and preventing the progression of dysphagia-related complications. The importance of early detection has been underscored by major organizations such as the American Stroke Association and the Joint Commission on Accreditation of Healthcare Organizations (JCAHO), which have highlighted that formal screening can reduce the incidence of pneumonia among hospitalized stroke patients [4].

Globally, the assessment of dysphagia varies significantly, reflecting differences in healthcare systems, resources, and clinical practices. In many developed countries, dysphagia is typically evaluated using a combination of clinical bedside assessments and advanced instrumental evaluations, such as the fiberoptic endoscopic evaluation of swallowing (FEES) and videofluoroscopic swallowing study (VFSS) [4]. These sophisticated diagnostic methods provide detailed insights into the swallowing process and are critical for guiding treatment. Additionally, bedside tools like the Mann Assessment of Swallowing Ability (MASA) and the Modified Barium Swallow Study (MBSS) are widely utilized to screen and categorize dysphagia severity and assess aspiration risk.

In resource-limited settings, however, the assessment of dysphagia often relies more heavily on clinical judgment and basic screening tools due to restricted access to advanced diagnostic technologies. In these contexts, standardized assessment tools are essential for ensuring consistent and reliable evaluations, which are crucial for effective dysphagia management worldwide. The MASA is particularly noteworthy for its simplicity, comprehensiveness, and ease of administration, making it a valuable tool not only in research but also in clinical practice across diverse healthcare environments.

Originally developed by Mann in 2002, the MASA is a bedside tool specifically designed to evaluate dysphagia in patients during the acute stage of stroke. It includes 24 items across four domains: (I) General Patient Examination; (II) Oral Preparation; (III) Oral Phase; and (IV) Pharyngeal Phase. Each item is scored on a 5- or 10-point scale, with a maximum score of 200 points, allowing clinicians to classify dysphagia severity and aspiration risk as nil, mild, moderate, or severe [5,6,7]. The MASA has been validated in multiple languages, including English, Belgian, Turkish, and Chinese, and has been applied to various conditions such as stroke, psychiatric disorders, oral cancer, brain injury, and pneumonia in the elderly [8,9,10,11,12,13,14,15,16].

Despite being the most widely used tool globally for bedside dysphagia assessment, the MASA is not yet available in Italy due to the lack of a translation and cultural adaptation into the Italian language. Currently, the only tools available for dysphagia assessment in the Italian healthcare context are advanced diagnostic methods like FEES and VFSS [4]. While these methods are highly effective, they require significant resources, can be invasive or uncomfortable for patients, and are not easily accessible in all healthcare facilities. These limitations underscore the need for a reliable, easy-to-administer bedside tool like the MASA, which can be effectively used in various clinical settings, including those where advanced diagnostic instruments are unavailable. Validating and standardizing the MASA in Italian is therefore crucial to enable healthcare professionals to accurately assess and manage dysphagia in post-stroke patients, ensuring consistent, evidence-based care that aligns with international standards.

In light of the existing gap, the purpose of this study to translate, cultural adapt, and evaluate the reliability and validity the Mann Assessment of Swallowing Ability (MASA) for the Italian post-stroke population.

## 2. Materials and Methods

The MASA was translated from English to Italian using the “Translation and Cultural Adaptation of Patient Reported Outcomes Measures—Principles of Good Practice” guidelines [17].

### 2.1. Ethics Approval and Consent to Participate

All procedures were performed in accordance with the ethics standards of the responsible committee on human experimentation (institutional and national) and with the Helsinki Declaration of 1975, as revised in 2008. The “Sapienza” university ethics committees approved this study: Rif. 4816 Prot.03/01/2018; this research involves data provided without any identifier or group of identifiers, which would allow the attribution of private information to an individual. Informed consent was obtained from the study’s participants.

### 2.2. Translation and Cultural Adaptation

The first stage of adaptation involved forward translation. The original MASA was translated into Italian by two native English speakers and an Italian otorhinolaryngologist who was proficient in English. They created three independent literal translations. An independent native speaker of Italian, who had not participated in the forward translations, synthesized these translations. Using this temporary version of the questionnaire, three Italian translators then performed a back-translation into English without having seen the original version. The back-translated instrument was compared with the original to identify any discrepancies. To ensure cultural relevance, two Italian speech therapists and one otorhinolaryngologist, fluent in both English and Italian, reviewed the first Italian translation and revised and reformulated some items to align closely with the original. The expert committee’s role was to consolidate all versions and produce the pre-final version of the questionnaire for field testing.

### 2.3. Pre-Test (Cross-Cultural Validity)

The pre-final translated version of the MASA, as outlined by Perneger et al. [18], was administered to a small representative group of individuals to assess its cross-cultural validity. To avoid bias, the same professional administered the test to each participant twice, with a 24–48 h interval between administrations to ensure no clinical changes occurred. This process led to the finalization of the Italian version of the MASA (MASA-ITT).

### 2.4. Participants

A minimum sample size of 50 participants with acute stroke, admitted to the inpatient stroke and neurological department, was required for the study. Participants had to be at least 18 years old, have ictal vascular cerebrovascular lesions, and speak Italian as their primary language. Individuals interested in participating gave their consent prior to being included in two scheduled testing sessions. Patients with different diagnoses were excluded from the study. Eligible participants who met the inclusion criteria were informed about the study [19,20]; those who were interested provided their consent before participating in two scheduled testing sessions. The reliability and validity of the culturally adapted scales were evaluated according to the “Consensus-Based Standards for the Selection of Health Status Measurement Instruments” (COSMIN) checklist. [21].

### 2.5. Reliability

The internal consistency of the MASA-IT was assessed using Cronbach’s alpha (α) to evaluate the scale’s homogeneity by measuring the interrelatedness of the items. According to Nunnally [22], an α coefficient of at least 0.7 is recommended for a new questionnaire to indicate satisfactory homogeneity among the items in the overall scale. In line with other studies on scale reliability, we also evaluated inter-rater and test–retest reliability.

The MASA-IT was administered to a sample of participants residing in Rome by three speech therapists. To assess inter-rater and test–retest reliability, the MASA-IT was first administered by three different professionals, and then again by one of these professionals after 48 h. The intraclass correlation coefficient (ICC) was calculated to measure both inter-rater and test–retest reliability, with an ICC of >0.70 considered indicative of stable test–retest reliability.

### 2.6. Validity

Concurrent validity was assessed using Pearson’s correlation analyses to determine the association between the MASA-IT and the Italian version of the two gold standard scales for the evaluation of dysphagia DOSS—Dysphagia Outcome Severity Scale—and Test De Pippo—Three Oz Water Swallow.

In the water swallow test, patients were given 3 oz of water and asked to drink from a cup without interruption. Coughing during or for 1 min after completion or the presence of a post-swallow wet-hoarse voice quality were scored as abnormal (DePippo et al. [23]).

The Dysphagia Outcome and Severity Scale (DOSS) is an easy-to-use 7-point scale designed to systematically assess the functional severity of dysphagia through objective evaluation. It provides recommendations on levels of independence, types of nutrition, and diet modifications (O’Neil et al. [24]). The scale was divided into seven independence levels: the first level was 7 that matches normal limits, 6 (modified independence), 5 (distant supervision), 4 (intermittent supervision), 3 (total supervision), 2 (maximum assistance), and 1 (dependent/non-per-oral nutrition). The scale was then divided into the two possible recommendations for nutrition and were linked to severity level: levels 7-3 (full oral nutrition) and levels 2-1 (nonoral nutrition). For each severity level that allowed oral intake, diet modifications were then added: levels 7-6 (normal diet consistency), level 5 (may need one diet consistency restriction), level 4 (one to two diet consistency restrictions), and level 3 (two or more diet consistency restrictions) (O’Neil et al.) [24].

All statistical analyses were performed using IBM-SPSS version 27.

## 3. Results

Participants were recruited from September 2017 to March 2018 at Policlinico Tor Vergata through the departments of neurosciences and stroke units.

### 3.1. Pre-Test (Cross-Cultural Validity)

Cross-cultural validity was assessed with 50 participants in September 2017. The characteristics of these participants are summarized in Table 1: the mean age was 69.16 years with a standard deviation of 14.27 years, 38% were men, and 56% had right-side brain injuries, while 44% had left-side injuries.

To ensure the consistent interpretation of the Italian version of the scales, a pilot study was conducted. This involved 50 evaluations performed by three independent examiners. Each examiner completed the protocol separately and then compared results. During this process, the examiners discussed and refined the understanding of each item in the protocol. Based on their feedback, the formulation of several items was adjusted to improve clarity. The analysis revealed that the results from the different examiners were strikingly similar, indicating a high degree of uniformity in the interpretation of the scales. However, it was noted that some items needed further clarification to enhance their comprehensibility and applicability. In response to this feedback, we updated the terminology used in the protocol. Specifically, the terms “dyspraxia” and “dysphasia” were replaced with “apraxia” and “aphasia”, respectively, to better align with common clinical language. Additionally, we incorporated illustrative examples to clarify the meaning of these terms. These revisions were made to ensure that the Italian version of the scales is both accurate and easily understood, improving its usability in assessing dysphagia across different cultural contexts.

### 3.2. Participants

From 1 September 2017 to 30 February 2018, 78 participants with acute stroke met the inclusion criteria and agreed to participate in the study. The average age of the participants was 69.03 years, with a standard deviation of 14.63 years, reflecting a diverse age range within the sample. This broad age distribution highlights that the study includes a wide spectrum of older adults affected by stroke.

In terms of gender distribution, 43.6% of the participants were men, indicating a fairly balanced representation of both genders in the sample. Regarding departmental affiliation, 64% of the participants were from the stroke unit, while 36% were from the neurological department. This distribution underscores the study’s focus on patients receiving specialized care in stroke units, with a significant number also coming from a more general neurological setting.

The analysis of brain injury side reveals that 60% of participants had left-sided brain injuries, compared to 40% with right-sided injuries. This indicates a higher prevalence of left-sided brain injuries within the sample, providing important context for understanding the distribution of stroke-related damage and its implications for assessment and management.

### 3.3. Reliability

The Italian version of the Mann Assessment of Swallowing Ability (MASA-IT) demonstrated strong internal consistency, evidenced by a Cronbach’s α of 0.88 (*p* < 0.01). This high value indicates that the scale is consistent in measuring the same construct across its items. Additionally, the item–total correlations were positive, confirming that each item contributes meaningfully to the overall scale, as detailed in Table 2, which presents the Cronbach’s alpha for each item of the Italian MASA.

A randomized subgroup of 50 participants underwent assessments to evaluate inter-rater and intra-rater reliability. The MASA-IT exhibited excellent inter-rater reliability (Table 3), with an intraclass correlation coefficient (ICC) of 0.999 (*p* < 0.01). All item-level ICC values were exceptionally high, ranging from 0.999 to 1, indicating a near-perfect agreement between different raters.

Furthermore, the test–retest reliability of the MASA-IT was also robust, with an ICC of 0.983 (*p* < 0.01). This value, along with item-level ICC values ranging from 0.971 to 0.991, demonstrates the scale’s reliability over time and its consistency in repeated measures.

### 3.4. Validity

Concurrent validity was assessed using Pearson’s correlation analyses to determine the association between the MASA-IT and the Italian version of the two scales’ gold standard for the evaluation of dysphagia DOSS—Dysphagia Outcome Severity Scale—and Test De Pippo—Three Oz Water Swallow. The correlation between the MASA-IT and DOSS scale is direct (r = 0.949, *p* = 0.01). Conversely, the correlation between the MASA-IT and Test De Pippo is indirect (r = −0.783, *p* = 0.01), indicating that the MASA-IT has good concurrent validity.

## 4. Discussion

In this study, we developed an Italian version of the MASA (MASA-IT). This article details the translation and cultural adaptation process of MASA-IT for use with Italian stroke patients, as well as the evaluation of its reliability and validity. Participants were hospitalized in the stroke unit and neurological department.

The translation and linguistic adaptation were carried out in accordance with the “Translation and Cultural Adaptation of Patient-Reported Outcomes Measures—Principles of Good Practice” guidelines [17]. This process was facilitated under the guidance of the instrument’s developers and a panel of experts, ensuring that the original intent of the items was preserved. The reliability and validity of the culturally adapted scale were evaluated using the COSMIN checklist [21].

The final version results from linguistic changes due to cultural adaptations. For these reasons, it was considered appropriate to translate the terms “Dysphasia” and “Dyspraxia”. The terms “Aphasia” and “Apraxia” are mostly used in the case of adult patients with neurological damage.

To assess the reliability of the scale, we examined its internal consistency, which yielded a Cronbach’s alpha value greater than 0.8 for all items. This high level of internal consistency ensures the scale’s robustness and coherence among its components. Notably, these findings are consistent with previous validations of the MASA in other languages. For instance, the Turkish version of the MASA demonstrated similarly strong internal consistency, with alpha values ranging from 0.899 to 0.901 [9]. This alignment with other studies reinforces the reliability of the MASA and supports its use in clinical settings for the assessment of dysphagia in post-stroke patients. Furthermore, this study is the first in the literature to analyze the test–retest reliability of the MASA-IT, providing a significant contribution to its psychometric validation. The stability of individual measures over time was assessed by calculating the intraclass correlation coefficient (ICC), an index ranging from 0 to 1, which was applied to evaluate both the consistency of the scale across different time points (test–retest) and the agreement between different operators during the initial evaluation. Notably, our findings are consistent with those of a previous validation study that also analyzed the test–retest reliability of the MASA, further reinforcing the reliability of the MASA-IT for clinical use across diverse settings and with different operators [9,10].

The concurrent validity analysis through the De Pippo and DOSS scales demonstrates significant correlations with the MASA-IT, underscoring its validity in diagnosing the presence or absence of dysphagia. Interestingly, the relationships between MASA-IT and the two gold standard instruments exhibit distinct patterns. The MASA-IT and DOSS scales are directly proportional; a high MASA score, indicating absent or slight dysphagia, corresponds to a high level on the DOSS scale. In contrast, MASA-IT and De Pippo are inversely proportional; a high MASA score aligns with a low De Pippo score. For instance, during the water test, a patient without a cough or gurgling voice receives a score of 0 on De Pippo and a high score on MASA-IT, signifying absent or slight dysphagia. Conversely, a patient exhibiting a cough or gurgling voice scores 1 on De Pippo and receives a low MASA-IT score, indicating severe or moderate dysphagia.

These findings are consistent with previous research, such as the study that evaluated the MASA’s concurrent validity against the Fiberoptic Endoscopic Dysphagia Severity Scale (FEDSS) [8]. In that study, the MASA demonstrated strong concurrent validity, reflected by a high area under the curve (AUC) of 0.85 for dysphagia detection. Our study similarly affirms the MASA-IT’s reliability as a diagnostic tool for dysphagia, albeit through different concurrent measures. The consistency across these studies, despite using different reference standards, reinforces the robustness of the MASA tool in various clinical settings. Furthermore, while the previous study recommended new cutoff scores to optimize diagnostic accuracy, our findings emphasize the MASA-IT’s adaptability and reliability, even when compared to alternative diagnostic scales like De Pippo and DOSS.

### Limitations of the Study

This study has certain limitations. The study involved a small sample, including only patients with cerebrovascular lesions. Therefore, all patients with different pathologies have been excluded. Further validation of the MASA-IT is needed to examine a larger sample than this and include patients with different diagnoses and dependent diseases. Although further work is needed, this Italian version of the MASA holds promise as a clinical and research tool.

## 5. Conclusions

The development and validation of the Italian version of the Mann Assessment of Swallowing Ability (MASA-IT) mark a significant advancement in the clinical management of dysphagia among stroke patients in Italy. With the high incidence of stroke and its associated complications, particularly dysphagia, the introduction of a culturally adapted and validated assessment tool like the MASA-IT is crucial for enhancing patient care. This study confirms that the MASA-IT is not only a reliable and valid instrument but also one that is attuned to the linguistic and cultural specificities of the Italian patient population.

The MASA-IT addresses a critical need within the Italian healthcare system, particularly in settings where advanced diagnostic tools such as fiberoptic endoscopic evaluation of swallowing (FEES) and videofluoroscopic swallowing study (VFSS) are either unavailable or impractical. By providing a standardized, easy-to-administer bedside assessment, the MASA-IT empowers clinicians to detect and manage dysphagia early, potentially improving patient outcomes and reducing the healthcare costs associated with stroke-related complications.

This tool not only aligns with international standards but also fulfills the specific requirements of the Italian healthcare context, thereby contributing to enhanced patient care and more efficient dysphagia management across the country.

## Figures and Tables

**Table 1:** Demographic characteristics and total score of the pre-test population.

	Sample Test–Retest *n* = 50	Total Sample *n* = 78
Age (mean ± SD)	69.16 ± 14.27	69.03 ± 14.63
Gender men, *n* (%)	19 (38)	34 (43.6)
Department, *n* (%)		
Stoke unit	33 (66)	50 (64)
Neurological	17 (34)	28 (36)
Vascular Injury Brain *n* (%)		
Left side	22 (44)	47 (60)
Right side	28 (56)	31 (40)

**Table 2:** Item-total analysis: Cronbach’s alpha for each item of the Italian version of the MASA.

	Mean ± SD	Alpha Di Cronbach if Item Is Eliminated
Alertness	9.89 ± 0.450	0.882
Cooperation	9.76 ± 0.651	0.880
Auditory comprehension	8.82 ± 1.991	0.877
Respiration	8.95 ± 2.622	0.891
Respiratory rate (for swallow)	4.71 ± 0.708	0.875
Dysphasia	4.36 ± 1.104	0.875
Dyspraxia	4.30 ± 1.059	0.872
Dysarthria	3.96 ± 1.227	0.873
Saliva	4.82 ± 0.559	0.881
Lip scalp	4.32 ± 0.941	0.872
Tongue movement	8.16 ± 1.721	0.872
Tongue strength	7.89 ± 2.043	0.886
Tongue coordination	8.16 ± 1.515	0.874
Oral Preparation	9.13 ± 1.509	0.868
Gag	4.72 ± 0.645	0.876
Palate	9.66 ± 0.888	0.880
Bolus clearance	9.12 ± 1.414	0.871
Oral transit	8.53 ± 1.770	0.868
Cough reflex	4.83 ± 1.350	0.876
Voluntary cough	9.03 ± 1.566	0.873
Voice	8.03 ± 1.743	0.884
Trachea	10.00	0.883
Pharyngeal phase	9.43 ± 1.011	0.875
Pharyngeal response	8.70 ± 2.551	0.874

**Table 3:** Test–retest reliability.

	Test Mean ± SD		Retest Mean ± SD	ICC	95% IC Lower	95% IC Upper
Intra-Operator	179.06 ± 18.28		178.68 ± 19.22	0.98	0.97	0.99
Inter-Operator	Operator I Mean ± SD	Operator 2 Mean ± SD	Operator 3 Mean ± SD			
	179.06 ± 18.28	179.08 ± 18.39	179.18 ± 18.21	0.99	0.99	1.00

## Data Availability

The original contributions presented in this study are included in the article; further inquiries can be directed to the corresponding author.
